# Frictional resistance exerted by different lingual and labial brackets: an *in vitro* study

**DOI:** 10.1186/2196-1042-14-37

**Published:** 2013-10-18

**Authors:** Luca Lombardo, Weronika Wierusz, Dominique Toscano, Roberto Lapenta, Andrea Kaplan, Giuseppe Siciliani

**Affiliations:** Postgraduate School of Orthodontics, University of Ferrara, Ferrara, Italy; Deparment of Orthodontics, School of Dentistry, and Instructor, Department of Dental Materials, School of Dentistry, University of Buenos Aires, Buenos Aires, Argentina; Department of Dental Materials, School of Dentistry, University of Buenos Aires, Buenos Aires, Argentina

## Abstract

**Background:**

Although much has been written on the implications of friction generated between orthodontic archwires and labial brackets, information on lingual brackets is still limited. Hence, we set out to investigate the frictional resistance exerted by different lingual and labial brackets, including both conventional and self-ligating designs. The effect of various factors, namely bracket/base width, slot size, inter-bracket distance, and first- (*Θ*cI) and second-order (*Θ*cII) critical contact angles were evaluated and compared.

**Methods:**

A plaster model of a pretreatment oral cavity was replicated to provide 18 (9 upper and 9 lower) identical versions. The anterior segments of each were taken, and the canine and lateral and central incisors were mounted with either lingual (7th Generation, STb, New STb, In-Ovation L, ORJ) or labial (Mini-Mono, Mini Diamond, G&H Ceramic) brackets. Mechanical friction tests were performed on each type of bracket using a universal testing machine. The maximum force necessary to displace NiTi wires of two different diameters (0.012, 0.014) was measured, using both elastic and metal ligatures with conventional brackets.

**Results:**

The frictional force necessary to displace the wires increased as the diameter of the wire increased in all tested brackets (*p* < 0.01). Friction was significantly higher (*p* < 0.001) with elastic ligatures, as compared with metal ones, in all conventional brackets. In the lower lingual group, significantly lower friction was generated at conventional lingual New STb brackets (*p* < 0.01) and ORJ lingual brackets (*p* < 0.05) than at self-ligating In-Ovation L lingual brackets. A significant statistical correlation between (*Θ*cI) and friction was detected in the lower labial bracket group.

**Conclusions:**

Friction resistance is influenced not only by the bracket type, type of ligation, and wire diameter but also by geometric differences in the brackets themselves.

## Background

Previous studies have emphasized the importance on the influence of the various mechanical properties that characterize orthodontic materials on friction [1–6]. Although the levels of friction generated between labial brackets and archwires have been described, information on the frictional behavior of lingual brackets is still very limited [7–14]. Frictional resistance (FR) has been attributed to many factors, such as bracket type, wire size and alloy, method of ligation, contact angles, and slot size [3]. Kusy and Whitley provide us with a precise description effect of the critical angle on friction in both active and passive configurations, concluding that if the angle between the archwire and the bracket slot is less than the critical contact angle, only classic friction is influential, because binding [1, 15] and notching are non-existent in a passive configuration [16, 17].

Several studies have also been carried out to elucidate the causes and effects of resistance to sliding in the passive configuration. Wire alloy, bracket material, surface modification, and roughness have been investigated [2, 12, 18–27], revealing that as the angle between the bracket and the archwire increases, the clearance between the archwire and the bracket slot is reduced. In this situation, binding occurs and in turn influences resistance to sliding, creating an active configuration [15, 28]. The active configuration itself, however, has received considerably less attention.

Although similar to labial brackets, lingual brackets feature several differences in terms of dimensions and clinical features, and labial mechanics cannot be applied to lingual devices [29, 30]. As the lingual arch radius is significantly narrower than the labial, a smaller inter-bracket distance is required in the former, especially in the lower anterior area, where the difference is particularly pronounced [29–31]. Hence, almost all lingual brackets are single and have a narrower M-D width [13]. To offset the reduced inter-bracket distance, more resilient archwires must be used to provide adequate rotation and torque control [32]. To better suit these smaller, more resilient archwires and due to the limited space available, lingual brackets generally have a 0.018 slot [32].

Despite these differences, few studies have attempted to evaluate frictional forces in lingual brackets. That being said, Park et al. did measure the friction generated by cobalt-chromium, stainless steel, and B-titanium archwires in two different lingual brackets, ORM and FJT, using a novel pin and disk friction tester. They found that cobalt-chromium wires generate significantly higher friction values than stainless steel and B-titanium versions and that friction was significantly lower when tests were performed with artificial saliva rather than in the dry state [13]. More recently, a comparative *in vitro* study of lingual brackets showed that as wire size and second-order angulation increased, so did the friction generated at all tested brackets, and friction can be reduced in self-ligating In-Ovation L lingual brackets using round rather than rectangular stainless steel wires [14].

In order to provide a comprehensive overview of the topic, we set out to evaluate the friction generated in both active and passive configurations of different lingual and labial brackets using plaster dental models, and to identify any correlation between frictional behavior and slot size, type of ligation, archwire diameter, bracket width, inter-bracket distance, and both first- and second-order critical contact angles.

## Methods

**Figure 1:**
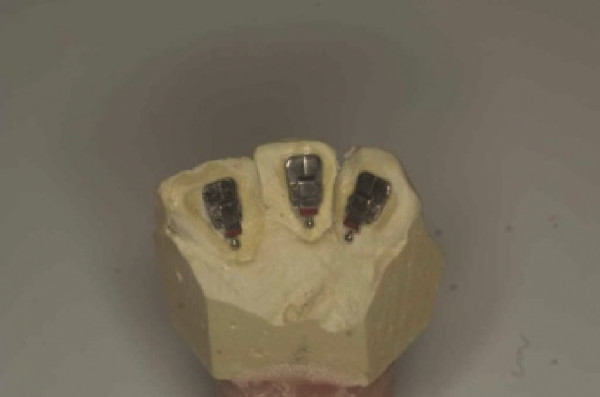
**Segmented plaster model with bracket positioned at halfway point of clinical crown.**

Eighteen plaster models (9 upper and 9 lower) were replicated from impressions of an untreated patient's oral cavity. The models featured a full set of fully erupted but misaligned permanent anterior teeth of normal shape and size, with no interproximal restoration, fractures, caries or age-related wear. No fractures or bubbles were present on the models, and crowding (Little's index) was no greater than 2 to 3 mm in order to limit potential notching between the archwire and bracket. Models of both the upper and lower arches were divided into segments featuring three teeth per sample: central incisor, lateral incisor, and canine (Figure 1). No distinction was made between the left and right segments. A total of eight commonly used orthodontic bracket types were tested, all with a 0.018 slot height, five lingual brackets namely In-Ovation L*, (DENTSPLY GAC International, Islandia, NY, USA), 7th Generation STb (Sybron Dental Specialties Ormco, Orange, CA, USA), New STb (Sybron Dental Specialties Ormco), ORJ lingual brackets (Hangzhou ORJ Medical Instrument & Materials, Hangzhou City, China), and STb (Sybron Dental Specialties Ormco) and three labial brackets namely Mini-Mono (Forestadent, St. Louis, MO, USA), Ormco Mini Diamond (Sybron Dental Specialties Ormco), and G&H Ceramic (G&H Wire Company, Greenwood, IN, USA). The lingual group included one example of a self-ligating bracket, indicated with an asterisk.

**Figure 2:**
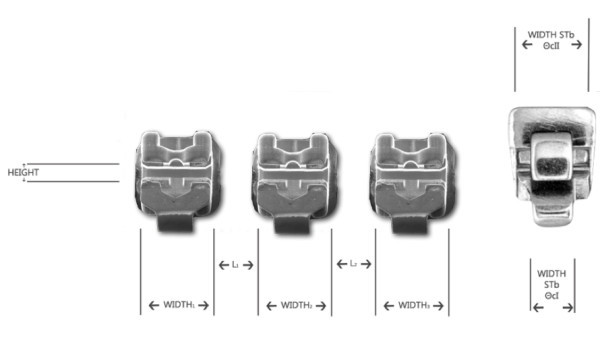
**Measurements of the width, slot height, and distance L.** Width (mean value for three distances W1, W2, W3). Distance between adjacent brackets (mean value for two distances L1, L2). Two different measurements for New STb bracket.

**Figure 3:**
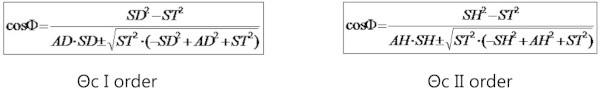
**Formulas for calculating first- and second-order critical contact angles.** SD, slot depth; ST, slot width; SH, slot height; AH, archwire size (for round wire, AH = AD).

All brackets were measured using an electronic gauge (Mitutoyo) and precision pins (Azurea) to obtain a precise mesiodistal bracket (slot) width, slot height, and distance between the two adjacent brackets (Table 1). Two bracket widths were measured for the New STb brackets, one as above (slot) and the other encompassing the two cleats in the mesial and distal parts of the slot. Only the maximum width was considered in the analysis. Likewise, two inter-bracket distances were measured for the New STbs, and only the smallest was considered (see Figure 2). The diameters of the two types of archwire were also measured using the same micrometer. The first- and second-order critical contact angles, *Θ*cI and *Θ*cII, respectively, were calculated as per the formulas shown in Figure 3[17].

**Table 1:** **Bracket groups, mean values of friction, width, inter-bracket distance, and first- and second-order critical contact angles**

Group	ID code	Bracket	Mean friction	Width	L	***Θ***cI	***Θ***cII
Lower lingual	A1	In-Ovation L	4.73	2.18	2.73	8.19	4.21
	A2	7th Generation	3.19	1.77	3.11	10.13	5.31
	A3	New STb	1.1	2.53/1.50	2.43/3.42	12.13	3.71
	A4	O.R.J.	1.79	2.28	2.72	7.80	3.91
	A5	OldSTb	2.32	2.04	2.82	8.77	4.50
Lower labial	A6	Forestadent	3.17	2.57	5.13	6.92	3.77
	A7	Ormco Mini Diamond	2.65	2.68	4.46	6.63	3.60
	A8	G&H Ceramic	4.64	2.83	4.35	6.26	3.41
Upper lingual	B1	7th Generation	4	2.36	3.48	7.56	3.91
	B2	O.R.J.	2.43	2.81	3.56	6.32	3.34
	B3	OldSTb	2.1	2.53	3.84	7.03	3.71
	B4	New STb	1.79	2.56/1.51	3.89/4.58	12.04	3.68
	B5	In-Ovation L	2.6	2.17	3.82	8.21	4.22
Upper labial	B6	Forestadent	3.27	3.55	6.30	4.99	2.68
	B7	Ormco Mini Diamond	2.62	3.49	6.07	5.08	2.71
	B8	G&H Ceramic	4.38	3.48	6.04	5.08	2.69

There were two different bracket widths for the New STb (Figure 2).

A total of 54 brackets were bonded in a clinically appropriate position, with the slot at the halfway point of the clinical crown, using a cyanoacrylate adhesive. Each typodont was inspected for general anatomical suitability before friction testing was performed. Two diameters of superelastic NiTi wire, 0.012 and 0.014 (G&H), both supplied in straight lengths, were tested. The ligatures used with conventional brackets were elastomeric modules (G&H) and SS ligatures (Preformed .010; G&H).

The frictional force was determined by means of a universal testing machine (INSTRON Corp, 1011, Norwood, MA, USA). Samples were placed in a fixed position, and wires were clamped to the machine. The force necessary to displace each wire was determined at a cross-speed of 1 mm/min. The wires were pulled in a distal direction in order to simulate the initial stage of alignment and leveling, when the archwires must slide through the brackets. The resistance of each bracket/archwire combination was tested in the dry state on each of the three teeth, and each measurement was performed in triplicate. Tests on conventional brackets were conducted with both elastic and metal ligatures.

### Statistical analysis

The bracket width, slot height, and archwire size were calculated as a mean of 10 measurements. To calculate the slot depth, we used the technical information supplied by the manufacturer. Due to their particular construction, we used two different measurements for the width and inter-bracket distance L for New STb brackets. In the subsequent statistical analyses, we used the mean values of *Θ*cI and *Θ*cII, and the mean width derived from three different brackets (central incisor, lateral incisor, and canine) for each bracket type.

The repeatability of measurements was assessed using ANOVA applied to the three friction values (each bracket/archwire combination was measured on each of the three teeth, and each measurement was performed in triplicate), and an *α* of 0.05. For statistical purposes we divided the study sample into four groups: Lower lingual (A1 to A5) Lower Labial (A6 to A9), Upper lingual (B1 to B5) and Upper labial (B6 to B9), as shown in Table 2.

**Table 2:** **Values of friction for different bracket and wire/ligature combinations and mean friction values**

Group	ID code	Bracket	Mean friction	Friction 0.012		Friction 0.014	
				Elastic	Wire	Elastic	Wire
Lower lingual	A1	In-Ovation L	4.73	3.55		5.90	
	A2	7th Generation	3.19	3.42	1.28	5.20	2.85
	A3	New STb	1.1	0.81	0.59	1.80	1.18
	A4	O.R.J.	1.79	1.86	1.72	1.77	1.82
	A5	OldSTb	2.32	1.73	1.48	3.40	2.65
Lower labial	A6	Forestadent	3.17	4.63	1.86	4.50	1.70
	A7	Ormco Mini Diamond	2.65	3.95	2.24	2.50	1.90
	A8	G&H Ceramic	4.64	4.80	1.07	8.10	4.61
Upper lingual	B1	7th Generation	4	4.45	2.24	7.00	2.29
	B2	O.R.J.	2.43	2.99	1.56	2.80	2.39
	B3	Old STb	2.1	2.34	0.76	2.90	2.39
	B4	New STb	1.79	2.01	0.67	2.37	2.10
	B5	In-Ovation L	2.6	1.67		3.52	
Upper labial	B6	Forestadent	3.27	4.53	1.63	5.50	1.40
	B7	Ormco Mini Diamond	2.62	3.84	0.62	4.00	2.03
	B8	G&H Ceramic	4.38	5.30	1.70	8.17	2.36

A linear mixed models test was used to analyze the friction within each of the four groups and to determine the impact of ligatures and wire size on friction. Pearson's correlation coefficient *r* was used to analyze the relationship between the critical angle and friction, width and friction, and inter-bracket distance and friction, taking into account the compatibility of the analyzed distributions with the parameters of normal distribution (Kolmogorov-Smirnov test). Finally, a linear mixed model was used to analyze the statistical effect of the brackets on friction, keeping other variables constant (wire and ligature).

## Results and discussion

### Results

Descriptive values of friction are reported in Table 2 as the means of each bracket/wire/ligation combination. The ANOVA test showed no statistically significant differences between the friction measurements (*p* > 0.05).

**Figure 4:**
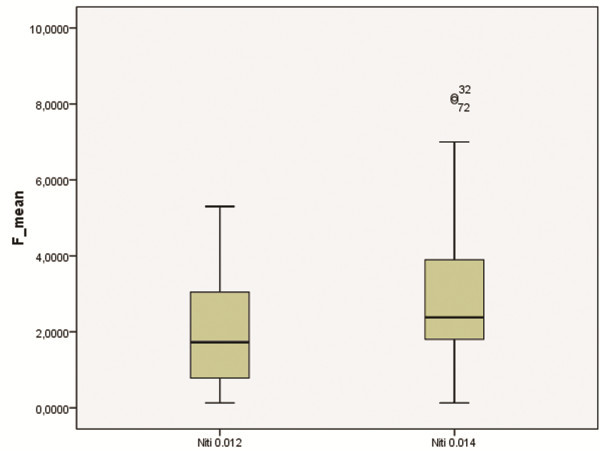
**Influence of wire size on friction.**

**Figure 5:**
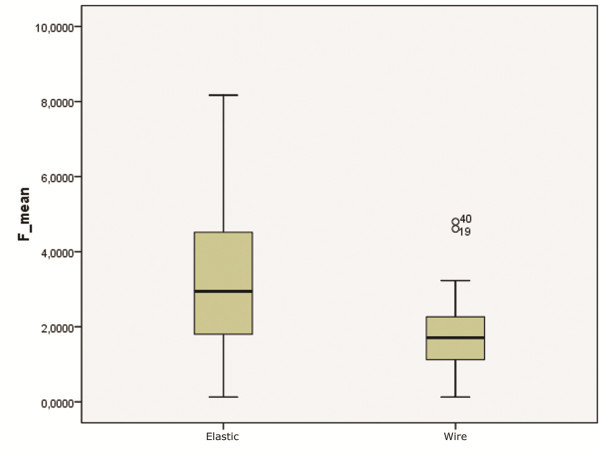
**Influence of type of ligation on friction.**

Wire dimension significantly influenced the sliding of the wire in all bracket types (*p* < 0.01) (Figure 4). The 0.014 wire generated higher friction levels for both the conventional and the self-ligating brackets. The method by which the wire was held in the slot also significantly influenced sliding. In all conventional brackets, elastic ligatures, as compared with metal ones, significantly increased friction (*p* < 0.001) (Figure 5).

**Figure 6:**
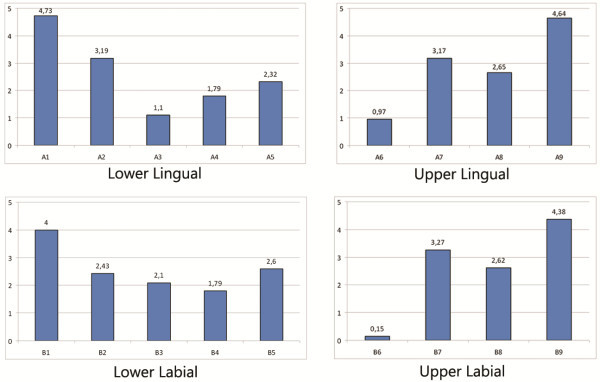
**Diagram showing friction values for all groups of bracket.**

Figure 6 shows the mean friction values for all groups. In the Lower lingual group, the conventional lingual brackets (A3, New STb, and A4, ORG) generated significantly lower friction (Bonferroni's post-hoc test *p* < 0.01 and *p* < 0.05, respectively) than self-ligating lingual brackets (A1, In-Ovation L) (Table 3). The self-ligating brackets also produced the greatest friction in the Upper lingual group, but neither this nor any other inter-group differences between brackets were significant.

**Table 3:** **Comparison of mean friction generated at lingual brackets (** ***p*** **< 0.05)**

Bracket (I)	Bracket (J)	Mean difference (I-J)	Std. error	Sig.	95% Confidence interval	
					Lower bound	Upper bound
A1	A2	1.5383333	.8037706	.749	-1.102888	4.179554
	A3	3.6308333^-^	.8037706	.004	.989612	6.272054
	A4	2.9333333^-^	.8037706	.024	.292112	5.574554
	A5	2.4091667	.8037706	.090	-.232054	5.050388
A2	A1	-1.5383333	.8037706	.749	-4.179554	1.102888
	A3	2.0925000	.8037706	.200	-.548721	4.733721
	A4	1.3950000	.8037706	1.000	-1.246221	4.036221
	A5	.8708333	.8037706	1.000	-1.770388	3.512054
A3	A1	-3.6308333^-^	.8037706	.004	-6.272054	-.989612
	A2	-2.0925000	.8037706	.200	-4.733721	.548721
	A4	-.6975000	.8037706	1.000	-3.338721	1.943721
	A5	-1.2216667	.8037706	1.000	-3.862888	1.419554
A4	A1	-2.9333333^-^	.8037706	.024	-5.574554	-.292112
	A2	-1.3950000	.8037706	1.000	-4.036221	1.246221
	A3	.6975000	.8037706	1.000	-1.943721	3.338721
	A5	-.5241667	.8037706	1.000	-3.165388	2.117054
A5	A1	-2.4091667	.8037706	.090	-5.050388	.232054
	A2	-.8708333	.8037706	1.000	-3.512054	1.770388
	A3	1.2216667	.8037706	1.000	-1.419554	3.862888
	A4	.5241667	.8037706	1.000	-2.117054	3.165388

Table 1 reports mean values of friction, width, inter-bracket distance L, and both first- and second-order critical contact angles. A negative statistical correlation was noted between *Θ*cI and friction in the Lower labial group (*p* < 0.05), indicating that the higher the mean angle, the lower the friction. Statistically speaking, a first-order critical contact angle was found to contribute to friction by 29%. No significant correlation was found in the other groups of brackets. In the Upper labial group, friction rose with increasing values of the width (*p* < 0.05) and decreasing values of inter-bracket distance L (*p* < 0.05). A similar tendency was observed for the inter-bracket distance L in both Upper and Lower lingual bracket groups (Table 4).

**Table 4:** **Correlation of width and inter-bracket distance L with friction**

Group		Mean friction	
		Mean width	Mean distance L
Lower lingual	Pearson correlation	.339	-.404
	Sig. (two-tailed)	.144	.078
	*n*	20	20
Lower labial	Pearson correlation	.306	-.164
	Sig. (two-tailed)	.249	.544
	*n*	16	16
Upper lingual	Pearson correlation	.179	-.405
	Sig. (two-tailed)	.451	.077
	*n*	20	20
Upper labial	Pearson correlation	.609^-^	-.606^-^
	Sig. (two-tailed)	.012	.013
	*n*	16	16

Significant correlation only in Upper lingual group; statistical tendency between distance L and friction in both lingual groups (*p* < 0.05); with an increase in the value of width, friction increases *r* = 0.61, *p* < 0.05 (*n* = 16); with a decrease in the value of distance L, friction increases *r* = -0.61, *p* < 0.05 (*n* = 16).

**Figure 7:**
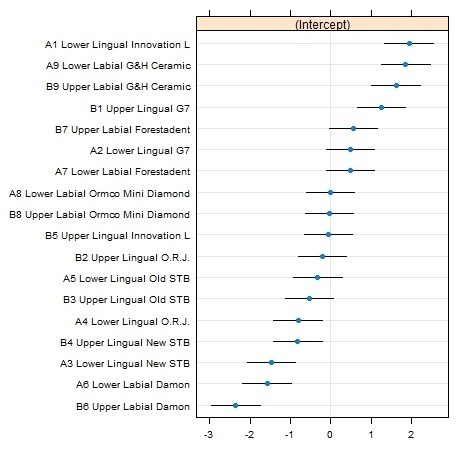
**Caterpillar plot: effect on friction for different types of brackets.** The straight lines represent the confidence intervals (CI) of the friction generated at each bracket, keeping the other variables constant (wire and ligature). If two different brackets have overlapping CIs, these brackets have a significantly different effect on friction.

The linear mixed model revealed that the different types of brackets had different effects on friction (Figure 7). In the Lower lingual group, keeping the other variables constant (wire and ligature), the In-Ovation L bracket produced higher values of frictional forces as compared to New STb.

### Discussion

Of the three types of labial brackets, in both the upper and lower arches, the ceramic (G&H) brackets generated the most friction, in line with that previously described by various authors [19, 21–34]. Also in line with previous findings [1, 3, 22, 35], we found that labial brackets generated far greater friction in the lower, with respect to the upper arch, except the upper bracket Mini-Mono (Forestadent), where the difference was almost imperceptible. The disparity between friction measurements for the upper and lower jaws can be explained by the fact that the lower jaw is considerably smaller than the upper, meaning inter-bracket distances are smaller, and rotation and torque are increased.

Examining the results for the lingual brackets, it is interesting to note that the values of friction were particularly high for the self-ligating brackets In-Ovation L with respect to the conventional brackets (Bonferroni's post-hoc test, *p* < 0.05), with the lowest values being found for STb brackets. Although little has been published on the issue to date, the only other study [14] to test the friction generated at lingual brackets, by Ortan et al., found very different results, namely that less friction was generated with self-ligating (In-Ovation L and The Magic), as opposed to conventional (Generation 7 and STb) brackets, in all bracket/wire/angulation combinations tested. However, these differences could be due to differences in study design. Indeed, Ortan et al. tested a single bracket mounted on a block, without considering the premolar dentition or other adjacent brackets. Furthermore, the three types of steel archwires examined (0.016, 0.016/0.016, and 0.017/0.025) are normally used in the final, rather than the initial stages of treatment. What is more, they focused on a bracket from a posterior segment, where there are no major differences between lingual and labial orthodontic techniques [29]. Moreover, they did not take into consideration the inter-bracket distance, a crucial factor in the lingual technique. Indeed, the anterior segment is always shorter in the lingual arch than that in the labial [31], and the inter-bracket distance will therefore be affected by different bracket sizes. This is particularly important in the anterior region, where the distances between adjacent brackets are very small, and should be a focus of any investigation into friction in the early stages of lingual orthodontic treatment.

Friction increased as archwire size increased when both conventional and self-ligating brackets were tested, and the smaller NiTi archwire (0.012) produced lower values than the larger (0.014) in all four bracket groups (*p* <0.01). These results are consistent with previous findings [2, 7, 20, 22, 36–38]. Unlike previous studies, however, we focused on round (nickel titanium 0.014 and 0.012), as opposed to rectangular wires to investigate the friction that develops in the first stages of treatment. Indeed, round small-diameter archwires are preferred in the alignment and leveling phase, where they are used to increase elasticity and minimize friction (sliding mechanics).

Several studies have previously described the increase in frictional force seen with decreasing values of *Θ*c [4, 5, 35, 39–42]. Nonetheless, these studies tended to focus on second-order *Θ*c, whereas we felt that it was important to look for increasing values of friction upon both vertical and horizontal displacement [43]. Indeed, our study revealed a negative statistical correlation (*p* < 0.05) between friction and *Θ*cI in the Lower labial group.

As expected from previous articles on the subject [36, 39–41], elastic ligatures generated greater friction (*p* < 0.001) than their metallic counterparts with both labial and lingual brackets.

The influence of bracket width on FR has already been described on several occasions, but, to our knowledge, only one of these studies evaluated this factor in the active configuration. Our finding that friction increases at greater bracket widths (upper labial group (*p* < 0.05) confirms the results of that study [1], which also set out to identify any correlation between FR and inter-bracket distance L [1, 26]. Once again, our results confirmed the previous finding that L is inversely related to friction, i.e., the latter increases with lower values of L, which could explain the negative statistical correlation (Upper labial group), and negative tendency (both lingual groups) we found between L and FR.

Our final aim was to determine whether the type of bracket had any statistically significant effect on friction. Keeping the other variables constant (wire and ligature), we found that this was indeed the case, a reminder to the clinician that bracket selection is crucial to the success of treatment in terms of anchorage control, particularly in extraction cases, due to the large arch length discrepancy in lingual orthodontics [1, 8, 22].

As far as we know, this is the first study to examine the effect of different lingual brackets bonded onto typodont and other factors on the friction generated between the archwires and slots in the initial stage of orthodontic treatment in an active configuration. Our findings have important implications on clinical sliding mechanics, but the study does have its limitations. In particular, rigid plaster models cannot mimic the physiological capabilities of the teeth *in vivo*, which would normally possess force-absorbing mechanisms. Nevertheless, typodonts are useful when assessing arch and tooth size limitation [8], and a previous comparison of *in vivo* and *in vitro* test values showed that laboratory and clinical frictional forces of immobile brackets are similar [44]. That being said, even though all brackets and archwires tested were precision measured, we only assessed one set of upper and lower brackets, meaning that we did not take into account the potentially significant intra-bracket variations that can occur [45–48].

This study looked at various factors that contribute to frictional force in different types of brackets, and it was not possible to establish the most influential factor. Nevertheless, significant differences in distance between the brackets mounted on the lingual surface especially in the lower arch shows that major factors may include the width of the bracket, although the present study failed to find a statistically significant correlation between friction and this factor in the lingual group. However, we did find a significant tendency, in both the upper and lower lingual groups, for frictional forces to increase with decreasing values of L, which is directly correlated to the width of the brackets. We also made the discovery that self-ligating brackets may not reduce frictional force in the lingual technique, something which needs to be investigated further to suggest improvements to future generations of lingual brackets.

## Conclusions

The type of bracket (self-ligating or conventional), bracket construction material, size of the wire, type of ligation, and geometric differences in the brackets all have an influence on the frictional force. However, more research into lingual brackets is required, particularly to confirm that frictional force is not reduced when lingual self-ligating brackets are used.
